# Service Evaluation of a Novel Menopause and Menstrual Health Service Embedded in a Secondary Care Mental Health Service With Interdisciplinary Input From Community Gynaecology.

**DOI:** 10.1192/bjo.2026.11539

**Published:** 2026-06-30

**Authors:** Lucienne Pullen, Segen Tecle, Sophie Behrman

**Affiliations:** 1 Oxford Health NHS Foundation Trust, Oxford, United Kingdom; 2 Oxford University, Oxford, United Kingdom

## Abstract

**Aims:**

Menopause is increasingly recognised as a contributor to mental health presentations. There is a lack of confidence amongst clinicians in investigating and managing menopause in psychiatric patients; up to 41% of psychiatrists reporting feeling “not confident at all” in responding to the hormonal health of female service users. We aimed to evaluate the acceptability and efficacy of a specialist service for menopause and menstrual disorders amongst patients in secondary mental health care.

**Methods:**

We devised and ran a Menopause and Menstrual Health Clinic offering email advice, case discussions, and assessments. The clinic is staffed by a consultant with special interest in the field, admin, and with MDT discussion input from Community Gynaecology. We have administered tailored teaching sessions to clinical services across the trust. Pre- and post-intervention survey data has been taken from service users, clinicians, and teaching recipients.

**Results:**

Initial feedback from teaching sessions shows improvement in knowledge and confidence (both 100%, n=12) and a positive impact on clinical practice (100%, n=10).

In the first eight months of operation, the clinic received 43 referrals from 37 referrers across primary and secondary care services. Early quantitative and qualitative feedback from patients and clinicians is positive. 100% of clinicians (n=9) who have responded to date find the clinic has improved the care they offer their patients, naming the “expert advice”, “comprehensive feedback”, and holistic reviews offered as key components of the clinic’s efficacy. 100% of clinicians and patients surveyed found the clinic easy to refer to or attend.Six-monthly follow ups from initial reviews are ongoing, but 100% (n=3) of patients surveyed to date have found treatment changes advised by the clinic to have had a positive impact on their mental health.

**Conclusion:**

Preliminary data suggests the clinic is beneficial to clinicians and patients, with strengths including discussion of novel treatment options, holistic assessment of needs, and open dialogue between professionals regarding hormonal symptomatology and management. Delivering teaching sessions for colleagues to introduce a hormonal lens to formulation and management plans further extends the reach and effectiveness of the clinic. Ongoing work is needed to embed this awareness in routine psychiatric practice, through the expansion of teaching provision and increasing the profile of the clinic’s criteria and referral process. Future research should consider the need to embed consideration of hormonal status into mental health assessments, as a step towards addressing inequitable health outcomes for women.”

